# Randomized double-blind controlled trial to assess the efficacy of intravenous acetaminophen associated with strong opioids in the treatment of acute pain in adult cancer patients: study protocol

**DOI:** 10.1186/s13063-022-06442-2

**Published:** 2022-07-06

**Authors:** Ofelia Leiva, Joel Castellano, Luz M. Letelier, Luis Rojas, Paola Viviani, Antonio Gonzalez, Pedro Perez-Cruz

**Affiliations:** 1 Departamento Medicina Interna, Facultad de Medicina, Pontificia Universidad Católica de Chile, Santiago, Chile; 2 Sección Medicina Paliativa, Departamento de Medicina Interna, Facultad de Medicina, Pontificia Universidad Católica de Chile, Diagonal Paraguay 362, Of 523, 8330077 Santiago, Chile; 3 Departamento de Salud Pública, Facultad de Medicina, Pontificia Universidad Católica de Chile, Santiago, Chile; 4 Departamento de Hematología Oncología, Facultad de Medicina, Pontificia Universidad Católica de Chile, Santiago, Chile; 5 Programa de Farmacología y Toxicología, Facultad de Medicina, Pontificia Universidad Católica de Chile, Santiago, Chile

**Keywords:** Acetaminophen, Opioids, Cancer pain, Inpatients

## Abstract

**Background:**

Cancer pain is one of the most frequent and relevant symptoms in cancer patients and impacts on patient’s quality of life. International and local standards recommend as an initial strategy the use of an analgesic scheme composed of strong opioids associated with adjuvants such as acetaminophen, based upon the assumption that combining drugs could have a better analgesic effect, could allow lowering opioid dosing, and could prevent the occurrence of adverse effects of opioids. However, there is uncertainty about the impact of acetaminophen as an adjuvant in patients who use strong opioids for moderate to severe pain management in cancer patients. The aim of this study is to assess the efficacy and safety of intravenous acetaminophen associated with strong opioids in hospitalized adult cancer patients who have moderate to severe cancer-related pain.

**Methods:**

We will perform a randomized double-blinded controlled study comparing intravenous acetaminophen 1 g 4 times a day versus placebo for 48 h as an adjuvant to strong opioids. We will assess pain intensity as a primary outcome, using the verbal numerical rating scale (VNRS, I0 to 10 scale with higher scores meaning higher pain intensity), and we will compare the mean difference in pain intensity between baseline and 48 h among the placebo and intervention groups. We estimate that a decrease of 1 point in the VNRS would be clinically significant. Assuming a standard deviation in pain intensity of 1.7 points, an alpha of 0.025, and a power of 0.8, we estimate a sample size of 112 patients, with 56 patients in each arm. Secondary outcomes include the difference in total opioid use between baseline and at 48 h among the groups, and adverse effects such as drowsiness, constipation, nausea, and vomiting would be evaluated.

**Discussion:**

The randomized, double-blind, placebo-controlled design is the best strategy to assess the efficacy of acetaminophen as an adjuvant in adult cancer patients with moderate to severe pain who are receiving strong opioids. We expect to contribute to national and international guidelines with these results.

**Trial registration:**

Clinicaltrials.gov NCT04779567. Registered on March 3, 2021. Retrospectively registered.

**Supplementary Information:**

The online version contains supplementary material available at 10.1186/s13063-022-06442-2.

## World Health Organization Trial Registration Data Set

**Table:** 

**Data category**	**Information**
Primary registry and trial identifying number	ClinicalTrials.gov NCT04779567
Date of registration in primary registry	10 June, 2019
Secondary identifying numbers	None
Source(s) of monetary or material support	Chilean National Grant for Research and Development in Health (Fondo Nacional de Innovación y Desarrollo en Salud - FONIS SA18I0039)
Primary sponsor	Agencia Nacional de Investigación y Desarrollo, Ministerio de Ciencia, Tecnología, Conocimiento e Innovación, Gobierno de Chile.
Secondary sponsor(s)	Facultad de Medicina, Pontificia Universidad Católica de Chile
Contact for public queries	OL, MD. +56 9 97180826; ofelia.leiva@gmail.com PPC, MD, MPH +56 9 66598073; peperez@uc.cl
Contact for scientific queries	OL, MD; PPC, MD, MPH. Pontificia Universidad Católica de Chile, Santiago, Chile
Public title	Randomized Double-blind controlled trial to assess the Efficacy of Intravenous Acetaminophen Associated with Strong Opioids in the treatment of Acute Pain in Adult Cancer Patients: Study Protocol
Scientific title	Randomized Double-blind controlled trial to assess the Efficacy of Intravenous Acetaminophen Associated with Strong Opioids in the treatment of Acute Pain in Adult Cancer Patients: Study Protocol
Countries of recruitment	Chile
Health condition(s) or problem(s) studied	Cancer related pain (moderate to severe)
Intervention(s)	Active comparator: *intravenous* Acetaminophen, 1 gr/100ml every 6 h during 48 h. Placebo comparator: saline 100ml
Key inclusion and exclusion criteria	Inclusion: Patients 18 years or older diagnosed with cancer, admitted to UC Christus Clinical Hospital. Patients reporting acute pain > or = a 4 in Verbal Numerical Rating Scale (VNRS) who are using strong opioids. Exclusion criteria: Altered mental status such as delirium. Patients presenting acute liver failure or chronic liver damage Child C. Patients with a history of allergies or hypersensitivity to acetaminophen. Patients imminently dying or with a survival prognosis of less than 72 hrs.
Study type	Interventional Allocation: randomized Intervention model: parallel assignment Masking: double blind (subject, investigator) Primary purpose: treatment
Date of first enrolment	June 6, 2019
Target sample size	112
Recruitment status	Recruiting
Primary outcome(s)	Difference in pain measured with VNRS between baseline and 48 h among the groups.
Key secondary outcomes	Difference in Morphine equivalent daily dose and side effects.

## Background

Cancer-related pain is a relevant clinical problem. During the last decades, the incidence of cancer has increased around the world [1] and pain is one of its most usual complications, with a frequency between 56 and 75% among advanced cancer patients [2, 3]. Uncontrolled pain has an impact on patients’ performance status, mood, appetite, sleep, and quality of life [4]. Fortunately, 90% of cancer pain can be treated effectively by following international guidelines such as the World Health Organization (WHO) analgesic ladder. This approach recommends that analgesic management should be staggered, depending on pain intensity. Although the WHO guideline was based on consensus rather than on evidence, it has become the standard of care at the international level and provides a framework for the gradual and systematic approach to the management of cancer pain [5–7].

Specifically, the WHO analgesic strategy proposes that for moderate (step II) to severe (step III) pain, an analgesic scheme based on strong opioids (morphine, methadone, or fentanyl, among others) should be established, associated or not with adjuvants, such as acetaminophen or non-steroidal anti-inflammatory drugs (NSAIDs) among others. This adjuvant scheme is justified on the basis of a possible additive or synergistic effect between drugs, since adjuvants have different mechanisms of action than opioids. The benefits of this association could allow an improvement in relieving cancer-related pain and could decrease the total opioid dose used per day. Also, as opioids are associated with relevant adverse effects such as drowsiness, nausea and vomiting, sedation, and constipation, among others, the addition of adjuvants could decrease the frequency and intensity of adverse effects with the consequent improvement of quality of life for this population [8, 9]. In addition to these considerations, acetaminophen is widely available, has a relatively low cost and an adequate safety profile, and also has a demonstrated usefulness for the management of pain of various etiologies, both orally and intravenously and particularly in postoperative pain, making it a drug that is easy to use with low risks [10, 11]. Studies have compared the effectiveness of oral acetaminophen versus intravenous acetaminophen in surgical patients and the results have shown analgesic equivalence [12] or a slight benefit towards IV acetaminophen [13]. The advantage of intravenous acetaminophen is that it can be used in patients with nausea, vomiting, anorexia, or an inability to take oral drugs for different reasons, which are frequent symptoms in cancer patients.

Looking at the downsides of this medication, the addition of acetaminophen implies the administration of an extra drug every 6 to 8 h either orally or intravenously adding to the burden that these patients experience. Also, despite the relatively low cost of acetaminophen, the wide extent of its use in some clinical contexts could imply an unnecessary increase in patients’ and health systems’ expenses.

In the context of cancer, acetaminophen is known to be effective in patients with mild pain [14]. However, it is uncertain whether adding acetaminophen to patients with moderate to severe cancer pain receiving strong opioids has any benefit. Interestingly, international practices are heterogeneous. For example, in Europe, patients with cancer-related pain who start strong opioids usually keep the administration of acetaminophen, while in North America it is discontinued [15, 16]. Prior studies on the effectiveness of acetaminophen as an adjuvant in cancer patients with moderate to severe pain are scarce and the majority have assessed its role in outpatients and patients with stable chronic pain. In an article recently published by our group, we assessed, using the Epistemonikos® methodology which analyzes different systematic reviews for a specific topic, whether the association of acetaminophen with strong opioids has any benefit for cancer patients with moderate to severe pain, such as improvement in analgesia, reduction in opioid requirements, or reduction in the frequency of adverse effects [17]. In this publication, in which five randomized studies were included that considered a total of 171 patients, we concluded that in cancer patients with moderate to severe pain: (a) adding acetaminophen to strong opioids could make little or no difference in pain control, with low certainty of the evidence; (b) it is not clear whether adding acetaminophen to strong opioids has any benefit on analgesic requirements in cancer patients, because the certainty of the evidence is very low; and (c) that it is not clear whether adding acetaminophen to strong opioids has any impact on cancer patients well-being, because the certainty of the evidence is very low [18–21]. A Cochrane systematic review also concludes that acetaminophen is not beneficial for pain management in this population [22, 23]. In summary, international guidelines recommend the use of adjuvants such as acetaminophen for patients with moderate to severe cancer-related pain, which is weakly supported by current evidence and which could add an unnecessary burden for patients and increase in costs for health systems.

These observations motivated our team to establish a single-center, prospective, two-group, placebo-controlled, double-blind, randomized study with the primary objective to assess the analgesic effectiveness of intravenous acetaminophen compared to placebo in cancer inpatients with moderate to severe pain who are receiving strong opioids. The analgesic effectiveness will be assessed by comparing pain intensity before enrolment and at 48 h after the intervention. Secondary objectives include assessment of the effect of acetaminophen in the reduction of opioids and in the frequency of opioid-related side effects.

## Methods/design

### Design

This is a randomized, controlled, double-blind, parallel-group, single-center clinical trial. This study received ethical approval from the Ethics Committee of the Pontificia Universidad Católica de Chile (ID #180328004). The study protocol was designed using the recommendations of the Consolidated Standards of Reporting Trials (CONSORT) statement [24] and according to the SPIRIT statement (checklist in Additional file 1) [25].

### Setting and partners

The setting of this study is the General Internal Medicine Ward of a tertiary-level university hospital (UC Christus Clinical Hospital) where patients will be recruited. The academic institution supporting is the Department of Internal Medicine - School of Medicine at Pontificia Universidad Católica de Chile. The statistical support was provided by an academic from the Department of Public Health at the same institution. This work is supported by a grant awarded by the National Commission for Scientific and Technological Research (CONICYT) from the Ministry of Education in Chile, through the National Grant for Research and Development Projects in Health (FONIS) during 2019 (ID number: SA18I0039).

### Participants

Inclusion criteria were patients 18 years old or older, diagnosed with cancer and admitted to UC Christus Clinical Hospital with moderate to severe pain (verbal analog scales >4), with any type of pain (somatic, visceral o neuropathic), who were using opioids or not before enrolment. Exclusion criteria included refusing to participate in the study; being unable to communicate in Spanish; having altered mental status; patients with acute liver failure or Child C cirrhosis, history of allergies, or hypersensitivity to acetaminophen; and patients imminently dying or with a survival prognosis of less than 72 h. Dropout criteria include patients who presented altered mental status or clinical decline during follow-up, patients who died during follow-up, or patients who withdrew consent during the study. The details of the inclusion, exclusion, and dropout criteria are described in Table 1.

**Table 1:** Selection criteria

**Inclusion criteria**	
Patients 18 years or older diagnosed with cancer	
Patients admitted to UC Christus Clinical Hospital	
Patients reporting acute pain > or = a 4 in the Verbal Analog Scale	
Patients may present with somatic, visceral, or neuropathic pain	
Patients with prior opioid use or virgin to opioids were eligible	
Patients may be users of NSAIDs or corticosteroids	
**Exclusion criteria**	
Patients who refuse to enter the study	
Patients who are unable to communicate in Spanish	
Altered mental status such as delirium	
Patients presenting acute liver failure or chronic liver damage Child C	
Patients with a history of allergies or hypersensitivity to acetaminophen	
Patients imminently dying or with a survival prognosis of less than 72 h	
**Dropout criteria**	
Patients who are unable to complete assessments due to altered mental status or clinical decline	
Patients who died	
Patients who withdrew consent throughout the intervention and follow-up period	

### Treatments

#### Opioid administration

**Fig. 1:**
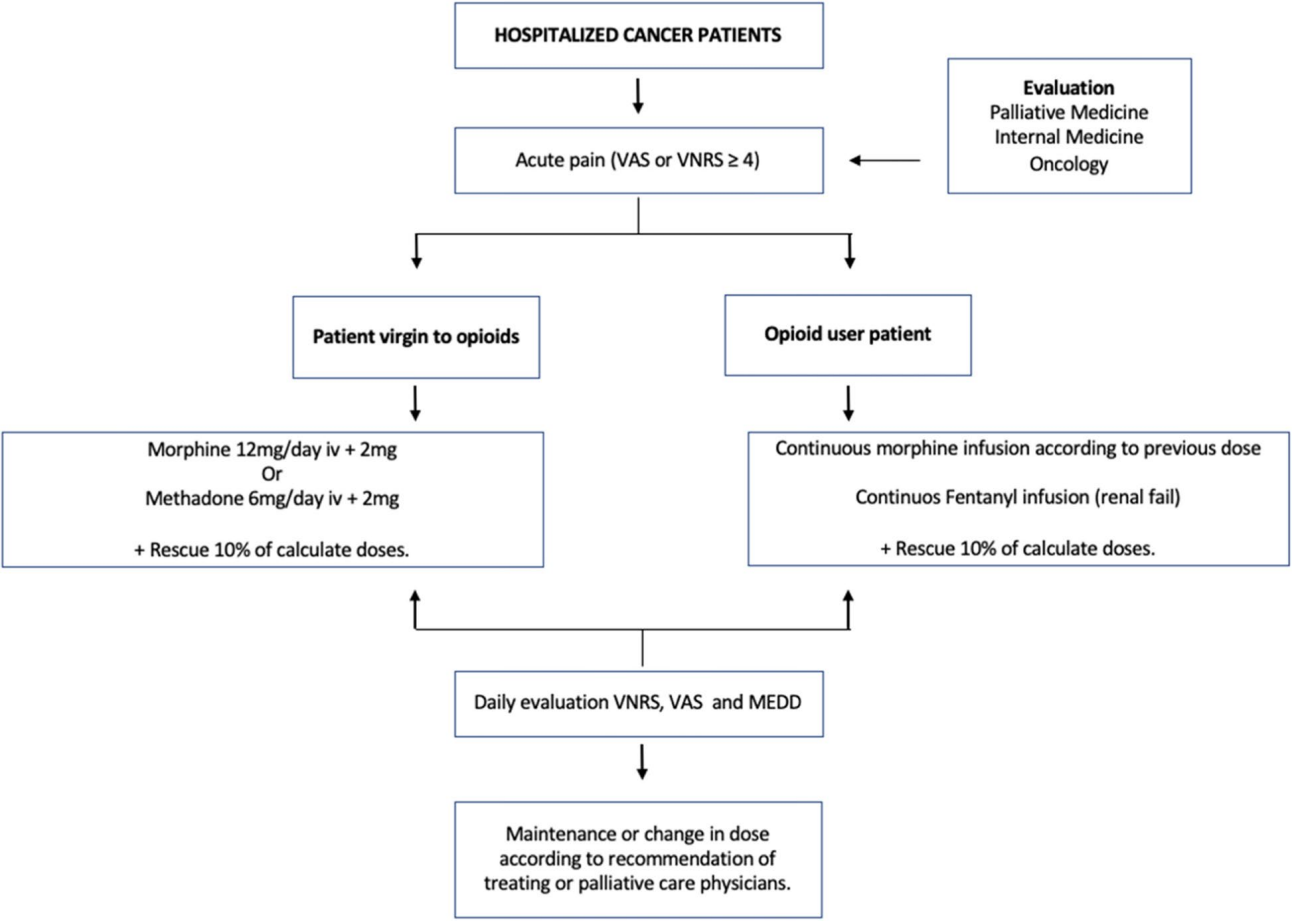
Standard analgesic protocol

Before starting the study, a standardized pain management protocol for cancer patients with moderate to severe pain will be implemented across the institution (Fig. 1). In this protocol, a standard analgesic protocol (scheduled strong opioids plus rescue doses, such as morphine, methadone, or fentanyl) will be started by the ward team upon admission in order to ensure that all cancer patients with moderate to severe pain will have an adequate analgesic scheme for pain control regardless study enrolment. Staff, fellows, and residents from the Internal Medicine, Oncology and Palliative Care (PC) teams will be trained in the implementation of this protocol (Fig. 1). Briefly, cancer patients with moderate to severe pain who are opioid naive will be started on scheduled morphine, methadone, or fentanyl by continuous infusion plus rescue doses. Standard doses will be recommended but dosing could be changed according to clinical judgement by treating clinicians. For patients with prior use of opioids, ward or treating clinicians could start scheduled methadone or morphine or fentanyl continuous infusion increasing the prior opioid dose. Early consultation to PC clinicians will be recommended for this population.

#### Acetaminophen-experimental group

Intravenous acetaminophen is usually delivered in a 100cc solution that is prepared in a transparent glass bottle. As the placebo cannot be prepared in the same type of bottle, the content of the acetaminophen preparation will be transferred to a standard 100cc plastic flask for IV infusions, which will be labeled with the name and ID number of the patient, with the drug to be administered labeled acetaminophen/placebo (including both names) and with the allocation number for the randomization. The preparation of the acetaminophen plastic flask will be indistinguishable from the placebo plastic flask. As the intervention will last 48 h, 8 plastic flasks with the drug will be sent directly to the clinical nurse in charge of the administration of the drug in the general ward.

#### Placebo-control group

The placebo will be prepared using the same plastic flask as used in the acetaminophen group. In the placebo group, it will be filled with 100cc of saline and will have the same label as the acetaminophen group; therefore, they will be indistinguishable from each other. In the case of the placebo group, 8 plastic flasks with the placebo will be sent directly to the clinical nurse in charge of the administration of the drug in the general ward.

#### Other treatments

As the research group wants to assess the impact of this intervention in the real clinical setting, treating clinicians will be allowed to be added according to clinical judgement. Non-steroidal anti-inflammatory drugs (NSAIDS), steroids, anticonvulsants, or other adjuvants could be added.

#### Monitoring of side effects during the intervention

Any adverse effects of opioids and acetaminophen will be monitored regularly at baseline and every 24 h for 48 h. In case of adverse effects, an independent investigator will assess on a case-by-case basis whether it will be appropriate to disclose patient allocation. In general, acetaminophen has a safe profile; however, opioids do present adverse effects more frequently.

### Recruitment and follow-up

#### Patient selection

All cancer patients admitted to the General Internal Medicine ward at UC Christus Clinical Hospital with moderate to severe pain who are using strong opioids will be eligible to participate. Screening will be performed by a trained nurse or health care professional who will actively seek for patients every morning among all admitted cancer patients with moderate to severe pain. Treating clinicians will be approached to ensure patient eligibility. The research nurse will approach the patient and will perform a screening instrument confirming inclusion and exclusion criteria. All patients meeting inclusion criteria and who are willing to participate in the study will sign an informed consent form that will be obtained by the research nurse.

#### Randomization

**Fig. 2:**
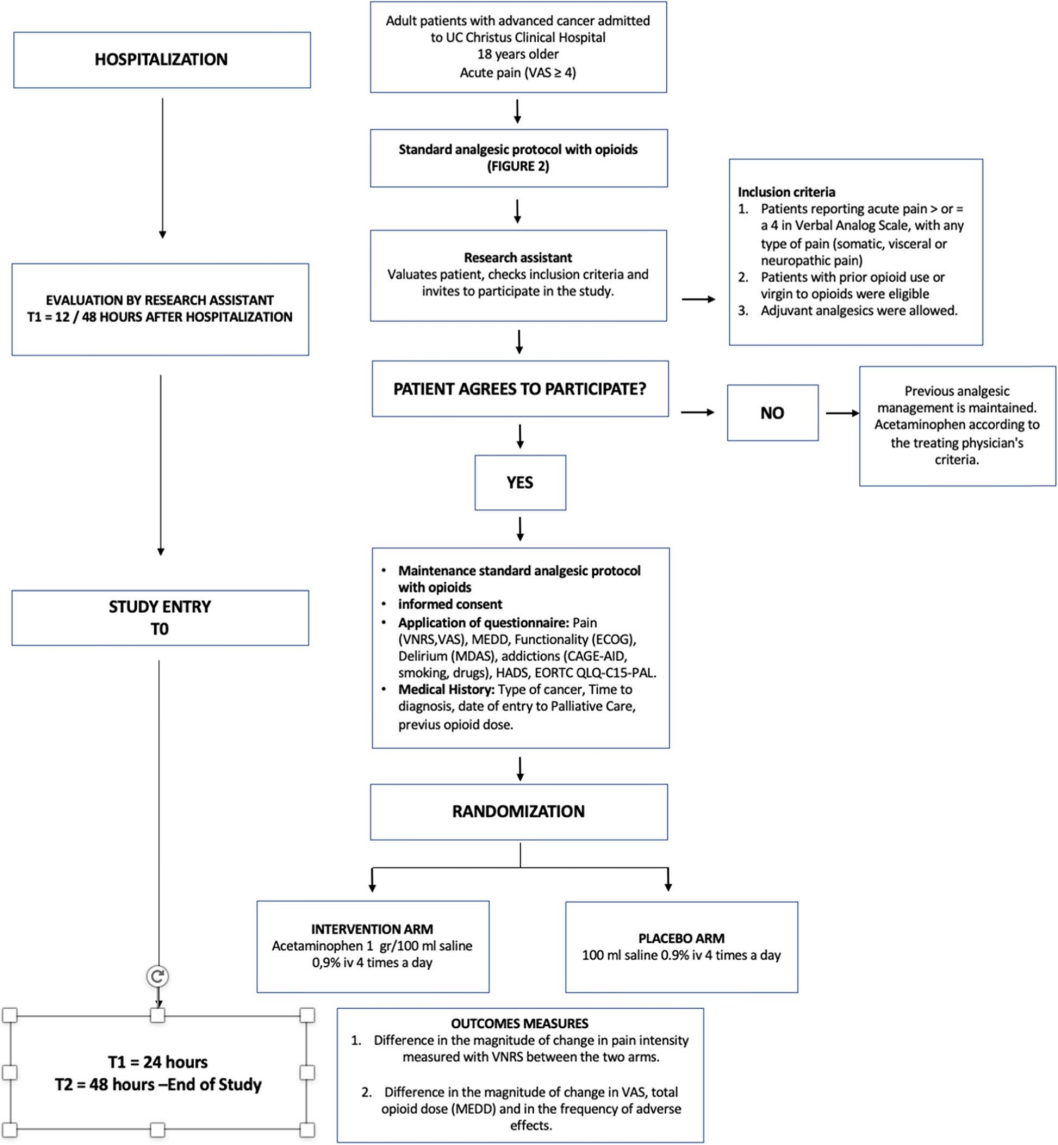
Consort diagram

After informed consent, eligible patients will be randomly assigned to either control (placebo) or experimental (acetaminophen) group with a 1:1 allocation (Fig. 2). The randomization procedure will be performed by the institution’s pharmacist using a web-based randomization software platform specifically designed to support data collection for research studies (Research Electronic Data Capture, REDCap®), a platform that provides automated export procedures for data downloads. The randomization will be performed following a stratified block randomization, with permuted blocks of 4 or 6 patients among which 50% of each block will receive placebo and 50% will receive acetaminophen to maintain the 1:1 allocation. The block sizes will not be disclosed to ensure concealment. The study will be blind with allocation concealment for all members of the research team, including principal investigators, data collectors, data analysts, clinical nurses, and patients, except for the institutional pharmacist. All research members will be unable to access the computer where the randomization is performed or the pharmacist’s allocation registry and will be unable to distinguish between the interventions (see below).

#### Implementation

Once the pharmacist has identified the allocated arm of the enrolled patient, a total of 8 identical plastic flasks will be prepared in the pharmacy service, with a total amount of 100ml of volume each and each one labeled as previously described. For arm A, IV acetaminophen will be prepared; for arm B, IV saline will be prepared. The eight plastic flasks will be delivered to the general ward and the clinical nurses will be in charge of administering the infusions during the 48-h study period. Precautions will be taken to ensure that treating physicians, clinical nurses, data collectors, data adjudicators, patients, and researchers will be blind to patient allocation, including the inability to access the allocation registry, and the similarity of the flasks and drug presentation. During the administration of the placebo/acetaminophen, clinicians will titrate pain medications following the recommendations of the standard analgesic protocol. As previously mentioned, clinicians will be able to make modifications of the standard analgesic protocol according to clinical judgement.

#### Follow-up and data collection

Patients will be asked to complete a baseline assessment questionnaire and then two other questionnaires at 24 and 48 h after enrolment. The questionnaires were selected seeking to assess the primary and secondary outcomes and variables that could impact patients’ pain experience. The study plan including the eligibility, the interventions and the assessments are described in Table 2. We included a variety of questionnaires and instruments, to assess eligibility criteria, the primary and secondary outcomes, and possible effect modifiers, including instruments to assess delirium (Memorial Delirium [MDAS]), pain (Verbal Numerical Rating Scale [VNRS] and Visual Analog Scale [VAS]), use of analgesia prior to admission, alcohol and drug consumption, risk of chemical coping, symptoms, psychological distress (Hospital Anxiety Depression Scale [HADS]) [28], quality of life, and symptoms associated with their current hospitalization [29]. Data will be registered in paper forms and will then be transcribed to a database, particularly designed to register research data (REDCap®). The principal investigator will randomly review the data registry in order to identify patterns for data errors and suggest recommendation to improve the data registry process. Data will be de-identified from the electronic database in order to maintain patient confidentiality throughout the study. Paper forms will be kept in a safety box that will be accessible only to data collectors and the principal investigator.

**Table 2:** Study plan — schedule of enrolment, interventions, and assessment

	Study period			
	Enrolment	Allocation	Post-allocation	Close out
	*-12h to 0*	0	*24h*	*48h*
**Enrolment**				
Eligibility screen	X			
Informed consent	X			
Standard analgesic procedure	X	X	X	X
Allocation		X		
**Interventions**				
Acetaminophen 1g/100ml Q6H IV		X	X	X
Saline 100ml Q6H IV		X	X	X
**Assessments**				
Demographic data	X			
MDAS	X		X	X
Type and location of pain	X			
VNRS	X		X	X
VAS	X		X	X
MEDD	X		X	X
Drug use in the past 24 h (corticosteroids, NSAIDs)	X		X	X
Cancer treatments received	X			
Karnofsky score	X			
CAGE-AID	X			
ESAS-SF	X			X
HADS	X			X
EORTC QLQ-C15-PAL	X			
Comorbidities	X			
Side effects	X		X	X
Patient perception of pain improvement			X	X
MCID			X	X

*MDAS* Memorial Delirium Assessment Scale, *VNRS* Verbal Numeric Analog Scale, *VAS* Visual Analog Scale, *MEDD* Morphine Equivalent Daily Dose, *CAGE-AID* CAGE Adapted to Include Drugs, ESAS-SF Edmonton Symptom Assessment Scale – Spiritual Distress, Financial Burden [26, 27], *HADS* Hospital Anxiety and Depression Scale [25], *EORTC QLQ-C15-PAL* [28] European Organization for Research and Treatment of Cancer QLQ-C30, *MCID* minimally clinical important difference

### Outcomes

Outcomes will be adjudicated by data collectors. In case any additional evaluation is required to adjudicate the outcome, precautions will be taken so that whoever does so also remains blinded.

#### Primary outcomes

The primary outcome of this study will be the difference in pain intensity between baseline (*T* = 0) and 48 h (*T* = 2) using the Verbal Numerical Rating Scale (VNRS) reported by the patient to assess the effects of the intervention in pain intensity. We will compare the difference in pain intensity between the groups. The VNRS is a tool in which the patient is asked to score the mean intensity of pain during the last 24 h in a scale from 0 to 10 with 0 meaning no pain at all and 10 meaning the worst possible pain. According to the score, pain is classified into no pain (score = 0), mild pain (score = 1 a 3), moderate pain (score = 4 to 6), and severe pain (score = 7 to 10). A patient is considered to have pain requiring therapeutic management if the patient reports a pain intensity of ≥ 4. Of note, this instrument has been recommended by WHO to assess pain in different settings [8].

#### Secondary outcomes

Another instrument used to assess pain intensity is the Visual Analog Scale (VAS) which will be used as a secondary outcome. The VAS uses a right triangle drawn on a paper, with a base of 10 cm wide and a height of 1cm on the right, in which its ends are delimited by a mark that expresses “without pain” on the left side and “worst pain I have ever felt” on the right side. The patient is asked to mark a vertical line crossing the horizontal line indicating the intensity of the pain. On the reverse, there is a superimposed line, with a graduation of 1 cm wide, which allows the data collector to identify the position in which the line marked by the patient is located. This indicates patients’ pain intensity score assigned by the patient on a scale from 0 to 10. We will also estimate the difference in pain intensity between baseline and 48 h using the VAS and compare the magnitude of the difference between the arms.

Another secondary outcome that we will use is the total morphine equivalent daily dose (MEDD) during the prior 24 h before the assessment comparing baseline (*T*=0) and 48 h (*T*=2). The MEDD represents the total dose of opioids used over the course of 24 h converted to an equivalent dose of parenteral morphine, following the standard equianalgesic conversion tables [26]. We will also compare the frequency of side effects (drowsiness, constipation, nausea and vomiting, allergy, and skin reactions among others) and the frequency of fever among the two arms during the study period.

Other secondary outcomes that will be assessed include MDAS to assess delirium, CAGE-AID to assess alcohol and drug consumption, the Edmonton Symptom Assessment Scale (ESAS-SF) to assess physical and psychological symptoms and financial and spiritual distress, the HADS to assess psychological distress, the EORTC QLQ-C15-PAL scale to assess the quality of life, and patient perception of pain improvement with a single question scale to assess the minimally clinical important difference (comparing the last 24 h with the 24 h before the beginning of the protocol, your perceive that: your pain has improved, has maintained the same, has worsened).

### Follow-up

The follow-up for efficacy outcomes will conclude after 48 h.

### Data collection and management

#### Sample size

To estimate the sample size, we decided to use the strategy of identifying the minimum clinically important difference (MCID) in pain according to the ESAS scale, which evaluates pain on a scale from 0 to 10, similar to the VNRS, which we will use as our main outcome. The MCID is defined as “the smallest change in a measurement that signifies an important difference in a patient’s symptoms” [27]. In a study conducted by Farrar et al. [30], the MCID for pain was defined as 2 points, which was evaluated in a short in-hospital follow-up period, a scenario that is similar to that of our study. In another study, conducted by Hui et al. [27], published in 2015, different methods for establishing MCID were evaluated. In that study, using the anchor-based method through the calculation of the receiver operating characteristic (ROC) curve, it was recognized that an improvement in pain intensity by 1 point on the ESAS scale was identified by patients as a clinically significant improvement, i.e., patients detect 1 point on the ESAS scale as an improvement in pain control, scale similar to the VNRS. In this study, the standard deviation for the pain score was 3 points, similarly to what was found in previous studies [27]. Using other similar strategies, a difference between 1 and 2 points was identified as clinically significant. However, in this study, the pain assessment was performed on an outpatient basis and with a 3-week difference between the first and the last assessment.

In an unpublished sample of 100 advanced cancer patients assessed in our PC unit, we found that the mean intensity of pain using the VNRS among patients with moderate to severe pain was 5.8 points with a standard deviation (SD) of 1.7 points.

From the data obtained from prior publications, considering an alpha of 0.025, with a power of 0.8, we estimated that a sample size of 112 patients would be required, with 56 patients in each group to detect a difference of 1 point in pain intensity between the groups, with a standard deviation (SD) of 1.7. These assumptions are supported by the following reasons:

- Because a difference of 1 point is considered to be what is clinically defined as significant, so we should try to detect a difference greater than that.
- Because we reported an SD of 1.7 in the initial pain scale in a sample of cancer patients in our unit.

#### Data analysis

For numerical variables, baseline characteristic data will be analyzed and represented with mean ± SD, or median with interquartile range (IQR), according to data type and distribution. Categorical variables will be described with frequencies and percentages. For bivariate analysis, Student *T*-test or ANOVA will be used for continuous variables given that our sample size is large enough to assume normal distribution of the averages by Central Limit Theorem. We will use Wilcoxon’s rank sum test to compare non-normally distributed data. Finally, multivariate mixed models will be adjusted for interest scores such as VNRS, VAS, and MEDDs at 48 h. As fixed effects in the model, we will consider de intervention (placebo or acetaminophen) and other factors (as tobacco) and the patient as the random effect. Subgroup analysis will be performed in this population, comparing differences in change of pain intensity according to the severity of pain and according to prior use of opioids. An intent-to-treat analysis will be performed for all relevant outcomes. A significance of 0.05 will be considered. The analyses will be performed using the statistical package STATA 14.0.

#### Data monitoring

In this study, the research team established that a Data Monitoring Committee (DMC) will not be required, given that no interim analysis is planned, patients included are in non-critical indications and will be treated for a relatively short time, and the drug under investigation is well characterized and known for not harming patients. Also, due to the short duration of the intervention and the small sample size established, no interim analysis will be performed and no external audits will be scheduled.

### Ethics

All protocol modifications will be reported to the Ethics Committee at Pontificia Universidad Católica de Chile and will be reported also in the *clinicaltrials.gov* registry. Given that in this case the intervention is of low risk, this project does not include compensation for participants.

## Discussion

Today, there is uncertainty whether or not acetaminophen is an effective analgesic in cancer patients with moderate to severe pain who are on strong opioids. The available evidence regarding this issue is scant and inconclusive. Answering this question is relevant to improve the quality of the care of this vulnerable population around the globe. We believe that this study will contribute with high-quality data to help elucidate the real role of acetaminophen as an adjunct to opioids in moderate to severe cancer pain. We expect that our findings will better support the analgesic interventions implemented for cancer patients so that national and international guidelines could be updated based on our results. To achieve this aim, we will use a randomized blind controlled study design, including cancer patients with moderate to severe pain in a way that makes it as inclusive as possible.

In this study, we decided to use intravenous acetaminophen, because it is possible to achieve greater control of the bioavailability of the drug given that it can be used in a wider variety of patients, even in patients with no oral intake. This will also facilitate the production of a placebo with similar external characteristics, thus achieving the blind. On the other hand, if the hypothesis that intravenous acetaminophen is not effective as an adjunct to strong opioids in moderate to severe cancer pain, it would be possible to homologate these results with oral acetaminophen. The results of this project will be shared with the community and local health authorities to make recommendations on acetaminophen in cancer patients with moderate to severe pain receiving strong opioids.

One strength of our proposal is that it will be carried out in a real clinical setting, allowing the use of other analgesic medications, which are often used in patient care. This will widen the applicability of the results. The study will be carried out in a single, highly complex academic inpatient center with an organized PC team that will ensure that patients will have access to the means and therapeutic alternatives to achieve good pain control. This may not include the reality of all health centers, affecting the generalizability of our results. It is important to highlight that the decision to make the study in the inpatient context will ensure that patients receive the medication as planned and will also contribute to early identify side effects or complications.

The main goal of this report is to provide a structured and detailed protocol in such a way as to avoid bias when analyzing the data and to be able to generate good quality evidence for decision-making in health.

## Trial status

Actively recruiting

Protocol version: III version, 09/28/2020

Date recruitment began: 06/10/2019

Date recruitment will be completed: 06/14/2021

## Supplementary Information

**Additional file 1.** SPIRIT checklist.

## Data Availability

The datasets used and/or analyzed during the current study will be available from the corresponding author on reasonable request.

## Abbreviations

CAGE: [Cut-annoyed-guilty-eye] tool to identify problems with alcohol consumption
CAGE-AID: CAGE Adapted to Include Drugs
CONICYT: National Commission for Scientific and Technological Research – Ministry of Education, Chile
CONSORT: Consolidated Standards of Reporting Trials
EORTC QLQ-C15-PAL: European Organization for Research and Treatment of Cancer QLQ-C30
ESAS-SF: Edmonton Symptom Assessment Scale – Spiritual Distress, Financial Burden
FONIS: National Grant for Research and Development Projects in Health – Ministry of Education, Chile
HADS: Hospital Anxiety and Depression Scale
IQR: Interquartile range
MCID: Minimally clinical important difference
MDAS: Memorial Delirium Assessment Scale
MEDD: Morphine equivalent daily dose
NSAID: Non-steroid anti-inflammatory drug
PC: Palliative care
ROC: Receiver operating characteristic
SD: Standard deviation
VAS: Visual Analog Scale
VNRS: Verbal Numeric Analog Scale
WHO: World Health Organization
